# Bax retrotranslocation potentiates Bcl-x_L_’s antiapoptotic activity and is essential for switch-like transitions between MOMP competency and resistance

**DOI:** 10.1038/s41419-018-0464-6

**Published:** 2018-03-22

**Authors:** Annika Hantusch, Kushal K. Das, Ana J. García-Sáez, Thomas Brunner, Markus Rehm

**Affiliations:** 1 Department of Biology, Chair of Biochemical Pharmacology, University of Konstanz, 78457 Konstanz, Germany; 2 Konstanz Research School Chemical Biology, University of Konstanz, 78457 Konstanz, Germany; 3 Interfaculty Institute of Biochemistry, Eberhard Karls University Tübingen, Hoppe-Seyler-Str. 4, 72076 Tübingen, Germany; 4 Department of Physiology & Medical Physics, Royal College of Surgeons in Ireland, Dublin 2, Ireland; 5 Centre for Systems Medicine, Royal College of Surgeons in Ireland, Dublin 2, Ireland; 6 Institute of Cell Biology and Immunology, University of Stuttgart, 70569 Stuttgart, Germany; 7 Stuttgart Research Center Systems Biology, University of Stuttgart, 70569 Stuttgart, Germany

## Abstract

The rapid, typically all-or-none process of mitochondrial outer membrane permeabilization (MOMP) constitutes a primary cell death decision that is controlled by the Bcl-2 family interactome. However, how strict all-or-none MOMP decisions are governed by and emanate from the dynamic interplay of pro- and antiapoptotic Bcl-2 family members remains incompletely understood. In particular, it is unclear to which extent the shuttling of Bcl-2 family species between lipid and aqueous phases contributes to regulating MOMP sensitivity. Here, we studied the interplay of tBid, Bax, and Bcl-x_L_, using a combined approach of deterministic mathematical modeling and retrospective as well as prospective experimental testing of model predictions. Systems modeling of the tBid–Bax interplay and their fluxes between cytosol and mitochondrial membranes reproduced experimental data on tBid-triggered Bax activation and oligomerization highly accurately. Extending these studies to analyze the cell-protective role of Bcl-x_L_ strikingly revealed that the activity of Bcl-x_L_ to retrotranslocate activated Bax from membranes back into the cytosol is essential to reproduce or correctly predict experimental outcomes. These included the potency of Bcl-x_L_ in suppressing Bax oligomerization, its role in limiting Bax membrane recruitment, the resistance threshold to low concentrations of MOMP triggers as well as a response potentiaton arising from combinations of tBid and sensitizer BH3-only peptides. Importantly, retrotranslocation activity of Bcl-x_L_ is necessary to strictly separate conditions of MOMP competency and resistance. Our results therefore identify Bax retrotranslocation by Bcl-x_L_ as an indispensable component of the molecular switch by which Bcl-2 family members govern cellular death decisions.

## Introduction

Pro- and antiapoptotic members of the Bcl-2 (B-cell lymphoma 2) protein family gather signals from stress sensing pathways and regulate the mitochondrial pathway of apoptosis^1,2^. The primary mediators of incoming stress signals are Bcl-2 family members with a single Bcl-2 homology (BH) domain, the so-called BH3-only proteins. The subgroup of activator BH3-only proteins, such as truncated Bid (tBid), Bim, or Puma, directly activate the effector Bcl-2 family proteins Bax and Bak^3,4^. These in turn oligomerize to form pores in the outer mitochondrial membrane, causing the release of cytochrome *c* and other proapoptotic factors into the cytosol^5^. This process of mitochondrial outer membrane permeabilization (MOMP) typically is an all-or-none event, resulting in the rapid and efficient activation of effector caspases and apoptosis execution^6,7^. Prosurvival family members, such as Bcl-x_L_, Bcl-2, and Mcl-1, efficiently antagonize both activator and sensitizer BH3-only proteins as well as Bax and Bak. Imbalances in the expression of Bcl-2 family members interfere with normal cellular homeostasis in multicellular organisms and can contribute to the complex etiologies of diverse degenerative and proliferative diseases^3,8,9^.

Importantly, the majority of critical interactions between Bcl-2 family members occur at or within the outer mitochondrial membrane. Membrane association and integration significantly affect protein conformations, binding affinities, and interaction patterns of Bcl-2 family members, so that authentic interaction data cannot be obtained from studies carried out solely in aqueous environments^10–12^.

Recently, Bax activation was described at greater mechanistic detail. Binding of the tBid BH3 domain to Bax unlatches the Bax core domain, thereby exposing the Bax BH3 domain and preparing Bax to form BH3-in-groove homodimers^13^. Strikingly, the lipidic environment at the mitochondrial outer membrane appears to facilitate the disengagement of core and latch domains and provides a surface to preorientate Bax for homooligomerization^13^. Bak is likely activated by a similar molecular mechanism in mitochondrial membranes^14^. Even though technologies such as scanning fluorescence cross correlation spectroscopy or fluorescence resonance energy transfer (FRET)-based assays in lipid environments now begin to provide reliable data from well-controlled experimental conditions^12,15,16^, obtaining a quantitative and kinetic understanding of the Bcl-2 interactome and MOMP regulation remains challenging^4,17^.

A further layer of complexity in the regulation of MOMP sensitivity might be added by the recently reported shuttling of Bcl-2 family members. Most prominently, it was demonstrated that the cytosolic fraction of Bax and Bax bound to the mitochondrial outer membrane exists in a dynamic equilibrium in healthy cells^18–20^. Bcl-x_L_, a predominantly membrane integrated family member, promotes retrotranslocation of Bax from the mitochondria into the cytosol and thereby limits Bax cytotoxicity^18,21^. However, to which extent retrotranslocation contributes to the antiapoptotic potential of Bcl-x_L_ remains undetermined so far.

Here, we studied the regulation of MOMP by the interplay of tBid, Bax, and Bcl-x_L_ at and within membranes, using a combined approach of deterministic mathematical modeling and experimental validation of model predictions. Bax retrotranslocation appears to be essential to provide MOMP resistance to residual, basal BH3-only protein stress, while still allowing switch-like efficient MOMP induction in response to activator BH3-only inputs across a narrow Bcl-x_L_ concentration range. Furthermore, sensitizer BH3-only proteins potentiate activators in their capacity to induce MOMP and we were able to quantify for the first time the contribution of Bax retrotranslocation to the overall antiapoptotic potential of Bcl-x_L_.

## Results

### Quantitative kinetic modeling of the tBid–Bax interplay accurately simulates Bax activation and oligomerization

We initially developed a core mathematical model of the tBid–Bax interplay at and within membranes to study if membrane recruitment, activation, and oligomerization of Bax, leading to MOMP, can be simulated authentically. This core model subsequently was used to analyze the potency of Bcl-x_L_ in preventing MOMP and to determine the contribution of Bax retrotranslocation to the antiapoptotic function of Bcl-x_L_. All processes were modeled using ordinary differential equations (ODE) (see the Methods section and Supplementary Material 1).

**Fig. 1:**
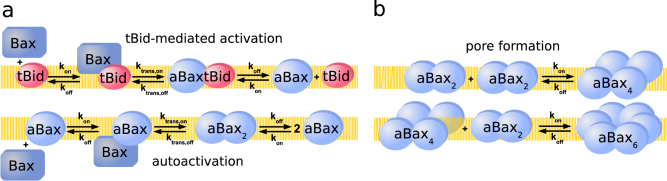
Molecular mechanisms of the tBid–Bax interplay and Bax pore formation captured in the core mathematical model. **a** Signaling processes providing active Bax species. Cytosolic Bax in its inactive conformation can be integrated into the membrane and activated either by interaction with tBid or with active Bax molecules (aBax) that already reside in the membrane. These processes were implemented as two-step reversible mechanisms, taking into account intermediate Bax species that are attached to but not yet fully integrated into the membrane. **b** Bax oligomerization and pore formation. Dimers of aBax were implemented to from higher order oligomers (tetramers, hexamers). Tetramers and hexamers were considered as a minimum requirement for pore formation

The activator BH3-only protein tBid was implemented to promote the insertion of monomeric Bax into the outer mitochondrial membrane^22^ (Fig. 1a). In the model, this process comprised serial reversible reactions, including tBid-mediated Bax membrane association and subsequent membrane insertion to yield Bax in its fully active conformation (aBax). aBax subsequently can form symmetric homodimers with other aBax molecules by BH3 domain/binding groove interactions^13^. In line with experimental evidence, aBax was assumed to recruit further Bax molecules to the membrane, thereby driving a Bax autoactivation loop^23–25^ (Fig. 1a). Since Bax autoactivation relies on the Bax BH3 domain^23^, the reaction sequence was implemented analogous to Bax activation by tBid (Fig. 1a). Experimentally, mostly even-numbered oligomers of aBax can be detected in membranes^26^. We thus modeled aBax oligomerization by assuming dimeric aBax species aggregating into tetramers (aBax_4_) and hexamers (aBax_6_) (Fig. 1b). Higher order oligomers were not explicitly modeled, since aBax_4_ and aBax_6_ appear to be sufficient for pore formation and fast cytochrome *c* release into the cytosol^27,28^. While Bax can continue to aggregate into higher order oligomers that are fluorescence microscopically easily distinguishable, these large clusters only form subsequent to MOMP^29^. The amounts of aBax_4_ and aBax_6,_ as a final output of the model, were interpreted to reflect the extent of membrane permeabilization (Fig. 1b).

**Fig. 2:**
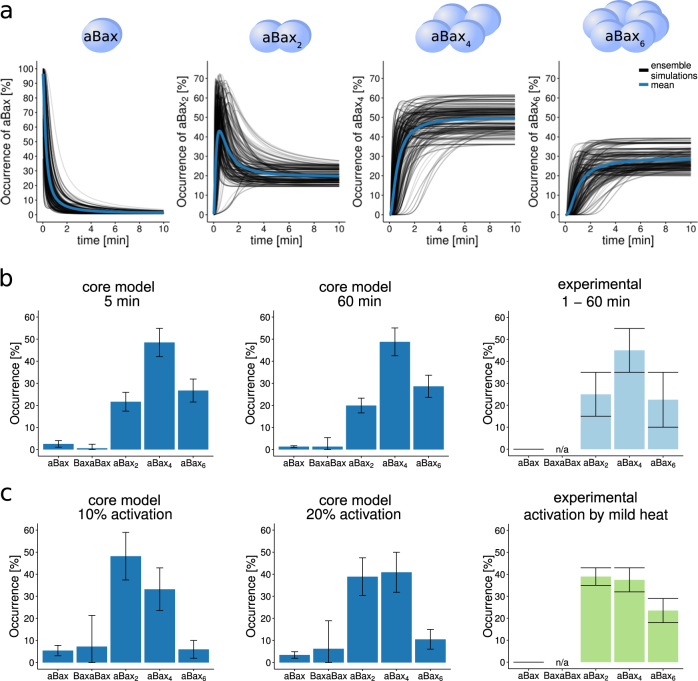
Ensemble simulations accurately reproduce tBid-induced Bax oligomerization kinetics and reliably predict Bax autoactivation. **a** Rapid oligomerization of Bax. Oligomerization kinetics are shown for an ensemble of 340 individual simulations. The mean kinetic is shown in blue. Input protein concentrations were 2.5 nM Bax and 5 nM tBid^26^. **b** Distribution of tBid-induced Bax oligomeric species obtained from the trained model at 5 and 60 min. Data are shown as mean and SD of the ensemble simulations. Quantitative experimental data were estimated from ref. ^26^ and are shown for comparison. **c** Distribution of Bax oligomeric species obtained by autoactivation. Model predictions are shown for inputs of 10 and 20% aBax. Experimental data as estimated from ref. ^26^ are shown for comparison and validation of predictions. Experimentally valid observations were assumed to be within the shown errorbars. Data are shown as means and SD

Bax multimers rapidly accumulate in membranes in response to tBid addition, as was demonstrated by measuring oligomerization kinetics in lipid bilayers^26^. However, the rate and dissociation constants for the underlying reactions and interactions so far can only be estimated within biologically plausible and justifiable parameter ranges. We therefore tested if model parameterizations could be obtained from these ranges that allowed us to reproduce experimental kinetics of Bax oligomerization. A detailed description of this procedure and the definition of suitable parameter ranges are provided in the Methods section and in Supplementary Material 1. Results from ensemble simulations (*n* = 340) using optimized parameter ranges demonstrate that oligomerization of membrane-bound aBax proceeds swiftly upon addition of tBid, with occurrences of aBax_4_ and aBax_6_ species rapidly reaching equilibrium within 5 min of triggering the reaction network (Fig. 2a), thus closely matching reported kinetics^26^. The distribution of oligomeric Bax species indicated that predominantly Bax tetramers and, albeit in lower amounts, Bax hexamers form. This distribution agreed well with the distribution of Bax oligomers measured experimentally (Fig. 2b).

Next, we used this core model to study the oligomerization of aBax resulting from autoactivation. To this end, we ran simulations in the absence of tBid and used small amounts of aBax as model inputs. For inputs of up to 10–20% aBax, we noted roughly equimolar amounts of aBax_2_ and aBax_4_ forming. To validate these predictions, we examined experimental data in which heat-activated aBax was used to initiate Bax oligomerization and pore formation in the absence of tBid^26^. Comparison of model predictions and experimentally observed distributions confirmed a close match of the results, with aBax_2_ and aBax_4_ being the predominant species (Fig. 2c). For conditions with high amounts of aBax inputs (80%), it would be assumed that oligomer distributions similar to those in the presence of tBid would be obtained. Control simulations indeed confirmed this assumption (not shown).

To conclude, our core model of the tBid–Bax interplay therefore reproduces experimental findings on tBid-induced Bax oligomerization, kinetically and quantitatively, and, without further modification of its structure or parameter values, accurately predicts Bax oligomer distributions obtained by autoactivation.

### Bcl-x_L_-mediated Bax retrotranslocation is critical for limiting Bax oligomerization

**Fig. 3:**
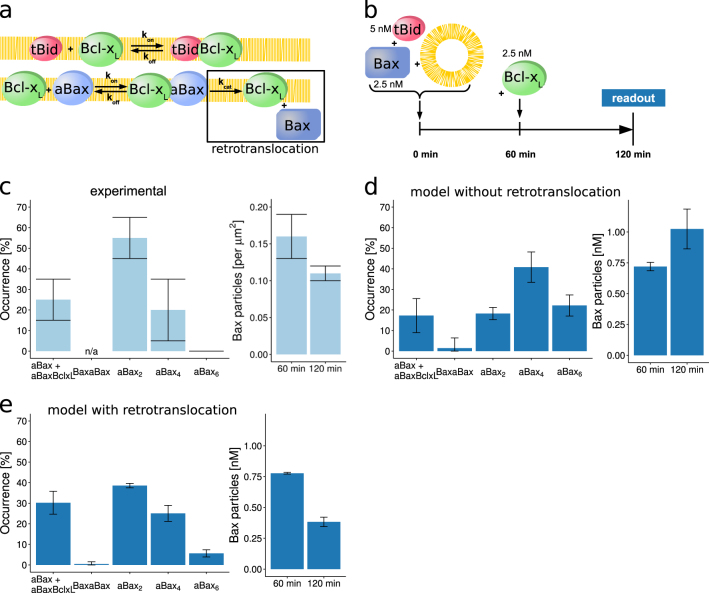
Retrotranslocation of Bax by Bcl-x_L_ is required to significantly impair Bax oligomerization. **a** Model extension by Bcl-x_L_. Bcl-x_L_ exerts its prosurvival function by binding to tBid as well as to active Bax monomers. The extension of the model by Bcl-x_L_ was implemented in two variants, including the retrotranslocation of mitochondrial Bax into the cytosol (black box) or not. **b** Experimental conditions for studying the influence of Bcl-x_L_ on Bax oligomerization, as described in ref.^26^. This scenario served as the reference for *in silico* studies. **c** Experimentally measured Bax oligomer distribution and Bax particle density at the membrane in the presence of Bcl-x_L_, as estimated from ref.^26^. Experimentally valid observations were assumed to be within the shown errorbars. **d** Bax oligomer distribution and concentration of membrane-bound Bax particles obtained from the model without Bcl-x_L_ retrotranslocation activity. Model predictions and additional model fitting approaches failed to replicate experimental data shown in **c**. Shown are simulation assuming rapid binding of Bcl-x_L_ to aBax and high affinity of the resulting complex (*k*_on_ 1 nM^−1^ s^−1^, *K*_D_ 0.1 nM). **e** Bax oligomer distribution and Bax particle concentration at the membrane obtained from the trained model with Bcl-x_L_ retrotranslocation activity. Experimental data shown in c can be reproduced. Data are shown as means and SD of ensemble simulations. Calculation of Bax particle concentrations at the membrane as described in Supplementary Table 3

We next integrated Bcl-x_L_ into the model to study the interplay of this classical triad of activator, effector, and prosurvival Bcl-2 family members, and to assess the potency of Bcl-x_L_ in preventing Bax pore formation in this signaling context. Bcl-x_L_ mediates its prosurvival function by at least two well-characterized mechanisms, i.e. (i) by binding to aBax and thereby preventing oligomerization and pore formation^30,31^, as well as (ii) by sequestering BH3-only proteins such as tBid^22,32^. We thus accounted for heterodimerization of Bcl-x_L_ with tBid and aBax in the extended model (Fig. 3a). Recent experimental studies revealed that Bcl-x_L_ also retrotranslocates aBax from mitochondrial membranes into the cytosol, a process that could add to the antiapoptotic potency of Bcl-x_L_^18,20,21^. We therefore implemented retrotranslocation as an optional model extension (Fig. 3a, black box).

Experimentally, the ability of Bcl-x_L_ to disassemble tBid-induced, pre-formed aBax oligomers can be quantified in lipid bilayers isolated from large unilamellar vesicles (LUVs)^26^ (Fig. 3b). Data on steady-state distributions of aBax oligomers and heterodimers with Bcl-x_L_ indicate that aBax hexamers cannot be observed upon addition of Bcl-x_L_ and that the majority of aBax resides within aBax_2_ and Bcl-x_L_-aBax species (Fig. 3c). Interestingly, simulations conducted with our core model, extended by the tBid-Bcl-x_L_ and aBax-Bcl-x_L_ interplay, failed to reproduce such data (Fig. 3d). Instead, we found that the majority of aBax still formed tetramers and hexamers (Fig. 3d). Even when searching a large parameter space, we failed to fit the model to the experimental data shown in Fig. 3c (see also control simulation provided in Supplementary Figure 1a). We next tested if the model variant that included the possibility for Bcl-x_L_ to retrotranslocate membrane-bound Bax into the cytosol was better suited to provide outputs that correspond to experimental findings. Indeed, results obtained with this model variant agreed very well with experimentally observed distributions of aBax oligomers when assuming a retrotranslocation rate of 0.05 s^−1^ (Fig. 3e).

In summary, these results demonstrate that the ability of Bcl-x_L_ to retrotranslocate aBax from membranes into the cytosol needs to be taken into account to reproduce experimental data on Bax oligomerization.

### Mathematical modeling accurately predicts limited Bax membrane recruitment in presence of Bcl-x_L_

**Fig. 4:**
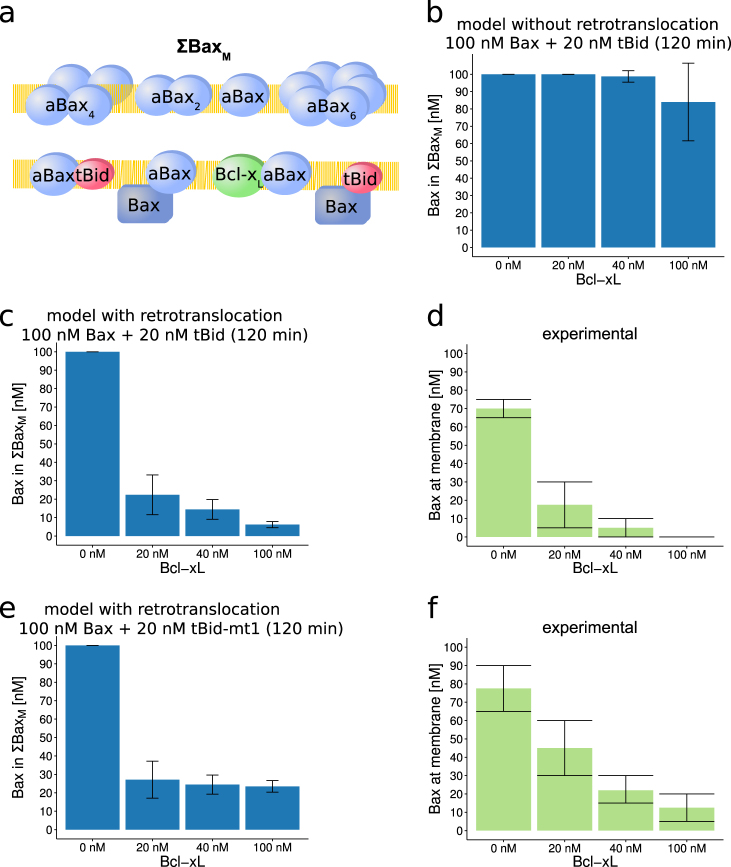
Systems modeling can accurately predict Bax membrane recruitment when taking Bax retrotranslocation activity of Bcl-x_L_ into account. **a** Definition of Bax membrane recruitment. As readout for Bax membrane recruitment, all Bax containing species residing at or in the membrane were considered (ΣBax_M_). **b** In the mathematical model lacking retrotranslocation activity, Bax translocation to membranes cannot be prevented. **c**, **d** The mathematical model including retrotranslocation activity of Bcl-x_L_ accurately predicts tBid-induced ΣBax_M_ at different concentrations of Bcl-x_L_ (**c**) when compared to the experimental data estimated from ref. ^33^ (**d**). **e**, **f** Model predictions for conditions in which a tBid variant was implemented that cannot bind to Bcl-x_L_. Predictions (**e**) correspond to trends observed experimentally as estimated from ref. ^33^ (**f**). Data are shown as means and SD from ensemble simulations or experimental data estimated from ref. ^33^, where experimental valid observations were assumed to be within the shown errorbars

We next used our model to estimate the overall amount of Bax recruitment to membranes in the presence of Bcl-x_L_. To this end, we studied conditions at which small amounts of tBid (20 nM) activate higher concentrations of Bax (100 nM), in the absence or presence of increasing amounts of Bcl-x_L_. To determine the overall recruitment of Bax, we took into account all Bax containing species at or within membranes (ΣBax_M_) (Fig. 4a). Without inclusion of Bax retrotranslocation, Bcl-x_L_ was not able to limit Bax membrane recruitment (Fig. 4b and Supplementary Figure 1b), even when assuming rapid association of Bcl-x_L_ with tBid and aBax, and high affinity of the resulting complexes (*k*_on_ 1 nM^−1^ s^−1^, *K*_D_ 0.1 nM). In contrast, ensemble simulations that took Bax retrotranslocation into account predicted that Bax membrane recruitment would be antagonized very potently already at concentrations of 20 nM Bcl-x_L_ (Fig. 4c). In comparison to previously reported experimental findings^33^, these predictions seemed to be highly accurate (Fig. 4d). We next eliminated the binding of tBid to Bcl-x_L_ in the model to study the contribution of this interaction to the potency of Bcl-x_L_ in limiting Bax membrane recruitment. For these conditions, higher amounts of Bax were predicted to accumulate at membranes, with high Bcl-x_L_ concentrations nevertheless efficiently limiting overall Bax recruitment to approximately 25% (Fig. 4e). Very similar trends were observed experimentally using the tBid variant tBid-mt1^33^, albeit with our predictions slightly overestimating the potency of Bcl-x_L_ at lower concentrations (Fig. 4e, f).

Overall, these findings demonstrate that the capability of Bcl-x_L_ to retrotranslocate Bax from membranes into the cytosol significantly contributes to its antiapoptotic potential and that mathematically modeling the tBid–Bax–Bcl-x_L_ interplay can accurately predict overall Bax membrane recruitment.

### Activator/sensitizer BH3-only potentiation can be predicted if retrotranslocation activity of Bcl-x_L_ is taken into account

**Fig. 5:**
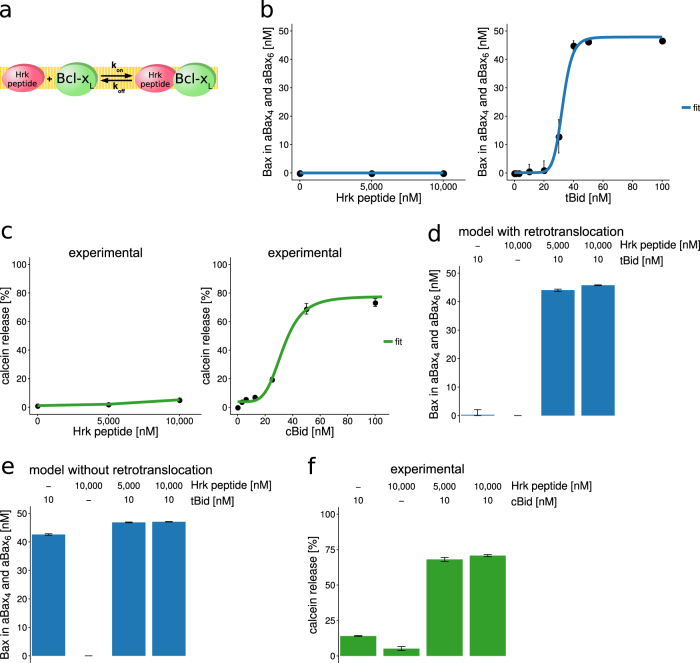
Mathematical modeling reliably predicts activator/sensitizer BH3-only synergies. **a** Model extension for inclusion of BH3-only sensitizer. The sensitizer was implemented to reversibly bind Bcl-x_L_, with kinetics as measured in ref. ^36^. **b** Bax oligomerization predictions in response to Hrk peptide or tBid. Starting conditions were 50 nM Bax and 20 nM Bcl-x_L_. **c** Experimental validation of model predictions. Dose–response curves of calcein release from large unilamellar vesicles after incubation with 50 nM Bax, 20 nM Bcl-x_L_, and varying amounts of Hrk peptide or cBid (cleaved Bid, consisting of tBid and a p7 fragment). **d** Prediction of Bax oligomerization for single or combined addition of tBid and Hrk peptide, when added to a system of 20 nM Bcl-x_L_ and 50 nM Bax. **e** Prediction of Bax oligomerization when not accounting for Bcl-x_L_ mediated Bax retrotranslocation. **f** Experimental validation of model predictions. Bax pore formation was experimentally determined by release of calcein from large unilamellar vesicles (LUVs). LUVs were incubated with 20 nM Bcl-x_L_, 50 nM Bax, and cBid and/or Hrk peptide as indicated. Data are shown as means and SD of ensemble simulations or experimental data

Sensitizer BH3-only proteins play major cell type- and tissue-specific roles in the regulation of apoptosis susceptibility^34^. We therefore studied how sensitizer BH3 peptides^35^ co-regulate Bax oligomerization together with activator BH3-only protein tBid, and how the retrotranslocation activity of Bcl-x_L_ influences Bax oligomerization in this scenario. For implementation into the mathematical model, we modeled a sensitizer Hrk peptide that binds to Bcl-x_L_ with published binding kinetics^36^ that cannot interact with or activate Bax (Fig. 5a). Consequently, Hrk peptide alone cannot induce Bax oligomerization (Fig. 5b). In contrast, a concentration of 40 nM tBid was sufficient to oligomerize nearly the entire pool of Bax (approx. 95%) (Fig. 5b). We next tested if these predictions could be confirmed experimentally by testing tBid and an Hrk-derived BH3 peptide^35^. For experimental validation of the model predictions, we determined the release of fluorescent calcein from LUVs, incubated with combinations of cBid/Hrk peptide, Bax, and Bcl-x_L_. In living cells and in *in vitro* assays, the processes of Bax oligomerization and release of proteins through Bax pores correlate reasonably well in time^22,29^. Calcein release from LUVs here therefore served as a surrogate marker or estimator for whether Bax pores form or not. As expected, release of calcein from LUVs only occurred when activator BH3-only protein tBid was added to Bax and Bcl-x_L_ (Fig. 5c). It was shown previously that Bcl-x_L_ alone or in combination with tBid can induce transient membrane permeability^32^. Experiments in which either Bax or Bcl-x_L_ and tBid or Hrk peptide were combined, respectively, confirmed that calcein release at conditions used in Fig. 5 can be solely attributed to the pore formation activity of Bax (Supplementary Figure 2).

Simulations with combinations of sensitizer and activator concentrations suggested that sensitizers would be expected to potently enhance tBid-induced Bax oligomerization and pore formation (Fig. 5d). Additional simulations predicted that potentiation can be expected as long as the retrotranslocation activity of Bcl-x_L_ is taken into account, since otherwise the reaction system was hypersensitive to residual amounts of activator BH3-only proteins (Fig. 5e, Supplementary Figure 3). Subsequent experiments confirmed these predictions, with combinations of suboptimal amounts of tBid and Hrk peptide (compare Fig. 5b, c) inducing efficient calcein release from liposomes (Fig. 5f). Taken together, these results therefore demonstrate that upon inclusion of retrotranslocation, our mathematical model can predict the experimentally observed, sensitizer-dependent potentiation of responses to activator BH3-only proteins.

### Bax retrotranslocation is essential to separate conditions of MOMP competency and resistance

**Fig. 6:**
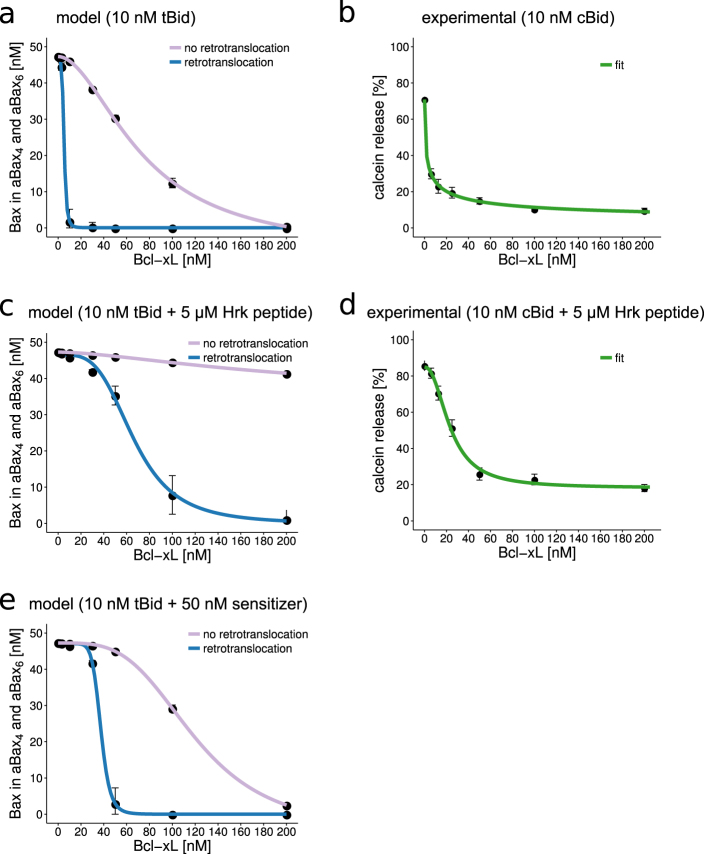
Retrotranslocation activity of Bcl-x_L_ is essential to strictly separate conditions of MOMP competency and resistance. Simulations of Bax oligomerization into pores (MOMP competency) in relation to increasing amounts of Bcl-x_L_. Simulations are performed for 50 nM Bax and 10 nM tBid (**a**) or 10 nM tBid plus 5 μM Hrk peptide (**c**) or 10 nM tBid plus 50 nM of a sensitizer with an affinity binding constant to Bcl-x_L_ of 1 nM (**e**). Blue lines refer to results from the model variant including retrotranslocation activity of Bcl-x_L_. **b, d** Experimental validation of model predictions. Bax pore formation was experimentally determined by release of calcein from LUVs. LUVs were incubated with 50 nM Bax and 10 nM cBid (**b**) or 10 nM cBid and 5 μM Hrk peptide (**d**) and concentrations of Bcl-x_L_ as indicated. Data are shown as means and SD of ensemble simulations/measurements

MOMP typically is a rapid, all-or-none cell fate decision to initiate the apoptosis execution phase^5,6^. The binary nature of death decisions implies that conditions of MOMP competency and resistance must be strictly separated to minimize the chance for an inefficient or submaximal induction of apoptosis execution^37^. We therefore studied to which extent this decision switch relies on the capacity of Bcl-x_L_ to retrotranslocate Bax into the cytosol. In these simulations, we steadily upregulated Bcl-x_L_ and calculated whether Bax oligomerization was inhibited. Our results demonstrate that as long as Bcl-x_L_ is capable of retrotranslocating Bax, conditions of complete Bax oligomerization and absence of Bax oligomerization are separated by a very narrow concentration range of sub-stoichiometric amounts of Bcl-x_L_ (Fig. 6a). In contrast, loss of retrotranslocation activity resulted in an approximately inversely proportional relationship of Bax oligomerization and the amounts of Bcl-x_L_, with super-stoichiometric amounts of Bcl-x_L_ being required to prevent Bax oligomerization and pore formation (Fig. 6a). As would be expected from model predictions for the retrotranslocation scenario, increasing Bcl-x_L_ concentrations efficiently suppressed calcein release from liposomes (Fig. 6b). We noted a similar threshold behavior when Hrk peptide contributions were taken into account in the model, albeit with the transition occurring over a somewhat broader Bcl-xL concentration range (Fig. 6c). Subsequent experiments confirmed this prediction (Fig. 6d). In absence of retrotranslocation activity, Bcl-xL instead would be expected to be incapable of preventing Bax pore formation (Fig. 6c). Of note, we used micromolar concentrations of Hrk peptide in our simulations and experiments, as it was shown previously that BH3 peptides are orders of magnitude less potent than the full-length proteins^36^, most likely due to decreased binding affinity of these peptides^38^. We also simulated conditions in which we assumed the presence of a bona fide sensitizer BH3-only protein. We implemented the sensitizer by using the association rate constant of the Hrk peptide but assuming a higher affinity to Bcl-x_L_. These simulations showed that nanomolar concentrations of sensitizer were sufficient to potentiate the activity of tBid and to maintain a sharp threshold separating MOMP competency and resistance (Fig. 6e). Based on the conditions studied here, we can estimate that retrotranslocation activity increases the antiapoptotic potency of Bcl-x_L_ at least 10-fold in the activator setting (20 vs. 200 nM to prevent oligomerization). Our findings therefore demonstrate that Bax retrotranslocation is essential to generate sharp decision thresholds with near-binary characteristics that separate MOMP competency and resistance.

## Discussion

**Fig. 7:**
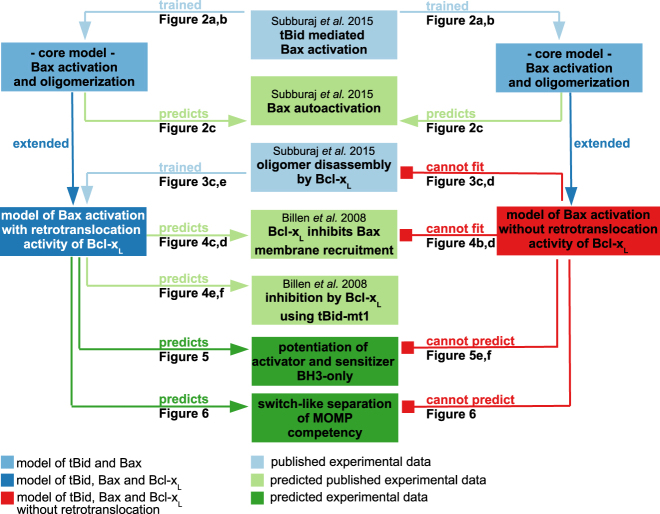
Flow chart providing an overview of model training, successful predictions, and experimental validation

Here, we studied the interplay of activator BH3-only protein tBid, multi-domain effector Bax, and their antagonist Bcl-x_L_, using a combined approach of mathematical systems modeling as well as retrospective and prospective experimental validation of model predictions (summarized in Fig. 7). The results of our simulations demonstrate that inclusion of Bax retrotranslocation by Bcl-x_L_ is indispensable for reproducing Bax membrane integration and oligomerization quantitatively and kinetically. Furthermore, the process of Bax retrotranslocation is essential for the MOMP decision to display near-binary, switch-like characteristics, with the signaling system transitioning from high MOMP competency to complete MOMP resistance across a narrow Bcl-x_L_ concentration range.

Even though Bax and Bcl-x_L_ have long been identified as key regulators of MOMP and apoptosis susceptibility^39,40^, evidence for continuous shuttling of Bax from mitochondrial membranes back into the cytosol emerged only in recent years^18,19^. For retrotranslocation to occur, Bax must interact with the hydrophobic groove of Bcl-x_L_ via its BH3 domain and additionally with Bcl-x_L_’s COOH-terminal membrane anchor, since preventing any of these interactions results in mitochondrial Bax accumulation^21^. Retrotranslocation can be observed in minimalistic, but well-controlled *in vitro* experimental settings, as evidenced by a decrease in Bax binding to the membrane in giant unilamellar vesicles upon Bcl-x_L_ membrane insertion^12^. However, within the complexity of living cells additional processes might undoubtedly play coregulatory roles. Mitochondrial specificity and membrane affinity for Bax may rely on additional cofactors such as VDAC2 (ref.^41^) and mitochondria–ER contact sites that seem to be preferential binding sites for Bcl-2 family proteins, probably through the local accumulation of Ca^2+^ and cardiolipin^42–44^. Cardiolipin indeed promotes tBid recruitment to membranes and efficient Bax activation^45,46^.

Retrotranslocation activity is not restricted to Bcl-x_L_, since other antiapoptotic family members, such as Bcl-2 and Mcl-1, retrotranslocate Bax at similar rates^18^. Effector protein Bak, closely related to Bax, likewise is retrotranslocated from mitochondria into the cytosol, albeit at far lower rates^20^. Overall, this indicates a continuous shuttling to and from mitochondrial membranes, including all major multi-domain Bcl-2 family members. Based on our results on the relevance of retrotranslocation, it is therefore likely that the continuous interplay of pro- and antiapoptotic fluxes establishes a steady state that prevents MOMP in stress-free scenarios. Indeed, replacing the C-terminal membrane anchor of Bax with that of Bak not only targets Bax to mitochondria but also reduces Bax retrotranslocation and is sufficient to trigger spontaneous MOMP and apoptosis execution^20^. In the multistep process of tBid-mediated Bax activation, measurements of early time points suggest that insertion of Bax into the membrane constitutes the rate-limiting step^22^. The fact that Bax undergoes a conformational change during membrane insertion further supports this notion^13^. At equilibrium conditions, the reaction rate of Bax translocation might therefore roughly equal the reaction rate of Bcl-x_L_-mediated Bax retrotranslocation. Indeed, training our initial model against legacy data provided very similar rates for these two processes (Supplementary Table 1).

Interestingly, it has been reported that Bcl-2 family members such as tBid and Bax can migrate between membranes in liposome assays, so that a stoichiometrically limited pool of Bcl-2 family proteins could nevertheless permeabilize larger amounts of liposomes in solution^47^. It could be relevant to take such processes into account in future stochastic, agent-based modeling approaches in order to better understand the spatiotemporal spread of MOMP as well as conditions in which MOMP is apparently confined to limited numbers of mitochondria^7,37^.

Another notable finding of our study is that retrotranslocation generates a signaling system in which conditions of MOMP competency and MOMP resistance are separated in a near binary, switch-like manner by modest changes in the amounts of Bcl-x_L_. Typically, all or no mitochondria in individual cells commit complete MOMP^6,7^. Conditions of submaximal or incomplete MOMP instead appear to be exceptions^37^. We furthermore show that sensitizer and activator BH3-only proteins can potentiate their activity to overcome the threshold for effective MOMP execution. Given a primed cell population with heterogeneous activator expression levels, the potentiating effect of a sensitizer in single cells can result in the observed synergistic effects on a population level. Synergies between sensitizer and activator BH3-only proteins were also described for various combination treatments in which signal transduction pathways culminate at the level of Bcl-2 family members, resulting in improved apoptosis responses of otherwise resistant tumors^48,49^.

The Bcl-2 family interplay and the control of the MOMP decision have been the subject of previous systems biological studies^50,51^. However, the detail at which the interactions were modeled varied greatly, and model development served different purposes. Since we demonstrated that retrotranslocation of Bax and, by extension, possibly Bak is crucial to strictly separate conditions of MOMP competency and resistance, it is tempting to speculate that including information on steady-state dynamics and shuttling rates of Bcl-2 family members might improve the prognostic power of translationally relevant systems models.

## Materials and methods

### Model implementation

The model was implemented as an ODE-based model in R (version 3.4.2). Supplementary Material 1 (Supplementary Figure 4 and Supplementary Tables 1–3) provides detailed information on all species, interactions, and rate constants. ODEs were integrated numerically using R’s routine lsoda (package deSolve, version 1.20), which provides an interface to the lsoda FORTRAN ODE solver, which switches automatically between stiff and nonstiff methods.

### Parameter estimates, sampling procedure, and model training

Biologically plausible parameter ranges were chosen as described in Supplementary Material 1. Ensemble simulations were performed as part of model training, using the following procedure. Biologically plausible parameter ranges were transformed to be log_10_ uniformly distributed in [0,1] and parameters were sampled from these distributions. We discretized the parameter space into a 20-level grid. Two hundred trajectories were sampled in parameter space following a previously described procedure^52^: In brief, for each trajectory a random grid point was selected. From this point one parameter was changed (increased or decreased) at a time by a value of *d* = 20/[2*(20−1)] until each parameter was changed exactly once. This provides a trajectory through parameter space with (*n* + 1) sampling points, where *n* is the number of parameters in the respective model used for the simulation. The choice of *d* as 20/[2*(20−1)] (for a 20-level grid) ensured that all levels have equal probability of being selected in the sampling strategy^52^. This resulted in an ensemble size of 200*(*n* + 1) model parameterizations. A function written in R generating trajectories in parameter space is provided as Supplementary Material 2.

For each output of interest (e.g. aBax [%]), dot plots of ensemble simulations were generated, where each dot corresponds to one simulation. This allowed us to assess the influence of each parameter on model outputs. Regions of the parameter space not providing outputs in agreement with experimental training data were excluded. Restriction of parameter ranges, simulation, and analysis of dot plots in an iterative procedure lead to the parameter ranges of the trained model (see also section on parameter ranges of the trained core and complete model in Supplementary Material 1, Supplementary Figures 5–31).

### Simulations and model predictions

Ensemble predictions were generated by sampling from parameter ranges of the trained models as defined in Supplementary Table 1 (Supplementary Material 1). Twenty trajectories in parameter space were generated for simulations shown in Figs. 2a, 5 and 6 and Supplementary Figures 2 and 3; 200 trajectories were generated for all other simulations. Associated protein amounts used as model inputs are listed in Supplementary Table 2 (Supplementary Material 1). All results were reproducible by independent resampling in parameter space.

### Peptides and proteins

Hrk peptide H-LRSSAAQLTAARLKALGDELH-OH was ordered with >95% purity from AnaSpec Inc. (Fremont, CA).

Purification of cleaved Bid, Bax, and Bcl-x_L_ was described by us previously^12^

### LUV permeabilization assay/Calcein release assay

LUVs of a size of approximately 100 nm were prepared, composed of 80% phosphatidyl choline and 20% cardiolipin. The dried lipid mixture was dissolved in buffer (20 nM HEPES, pH 7.4) and 80 mM calcein (fluorescein-bis-methyl-iminodiaceticacid at pH 7.5) was entrapped in lipid vesicles at a self-quenching concentration, so that its release into the external medium is accompanied by an increase in fluorescence intensity. To form the vesicles, the solution with lipids at a final concentration of 4 mg ml^−1^ was vortexed and passed through five cycles of freezing and thawing. The generated multilamellar vesicles were extruded >30 times with a 100 nm membrane filter (Avestin). LUVs were incubated with Bid, Bax, Bcl-x_L_, and Hrk peptide at room temperature. LUV concentrations in the final assays were estimated to lie within the range of 20–300 pM. The kinetics of calcein release were studied using a Tecan Infinite M200 microplate reader (Tecan, Switzerland).

The percentage of release *R* was calculated from:

$$R = (F_{\mathrm {S}} - F_0) \div (F_{\mathrm {max}} - F_0) \times 100,$$

where *F*_0_ is the initial fluorescence of LUVs; *F*_max_ is the maximum fluorescence after final addition of 5% TritionX-100; *F*_S_ is the equilibrium fluorescence following addition of Bcl-2 family proteins.

## Electronic supplementary material

Supplementary Material 1(DOCX 3214 kb)
