# Supplementary material for: Bax retrotranslocation potentiates Bcl-xL’s antiapoptotic activity and is essential for switch-like transitions between MOMP competency and resistance
Source: Cell Death Dis. 2018 Mar 22;9(4):430. doi: 10.1038/s41419-018-0464-6 (PMC5864878; doi:10.1038/s41419-018-0464-6)
Supplement: Supplementary file 1 — Supplementary Material 1(DOCX 3214 kb) [file 41419_2018_464_MOESM1_ESM.docx]

**Supplementary Material**

**Bax retrotranslocation potentiates Bcl-x_L_’s antiapoptotic activity and is essential for switch-like transitions between MOMP competency and resistance**

Annika Hantusch, Kushal K. Das, Ana J. García-Sáez, Thomas Brunner^#^, Markus Rehm^#^

^#^joint senior authors

This supplementary material contains

p.2 Supplementary Figure 1: Control simulations for models in Figures 3e and 4c

p.3 Supplementary Figure 2: Time courses of modeled Bax recruitment into pores and experimentally measured calcein release from large unilamellar vesicles in settings with either Bax or Bcl-xL

p.4 Supplementary Figure 3: Time courses for simulations and experiments presented in Figure 5b,c,d,e,f

p.5 Supplementary Figure 4: Overview of all modeled processes and numbering of model parameters

p.6 Information on prior estimates of parameter values

p.7 Supplementary Table 1: Parameter ranges for all simulations

p.8 Supplementary Table 2: Inputs and simulation time for all simulations

p.9 Supplementary Table 3: Definition of outputs for all simulations

pp.10-20 Supplementary Figures 5-16: Dot plots for all trained parameters of core model (inputs and outputs as in Figure 2a,b)

pp.21-36 Supplementary Figures 17-31: Dot plots for all trained parameters of complete model (inputs and outputs as in Figure 3e)

p.37 Bibliography


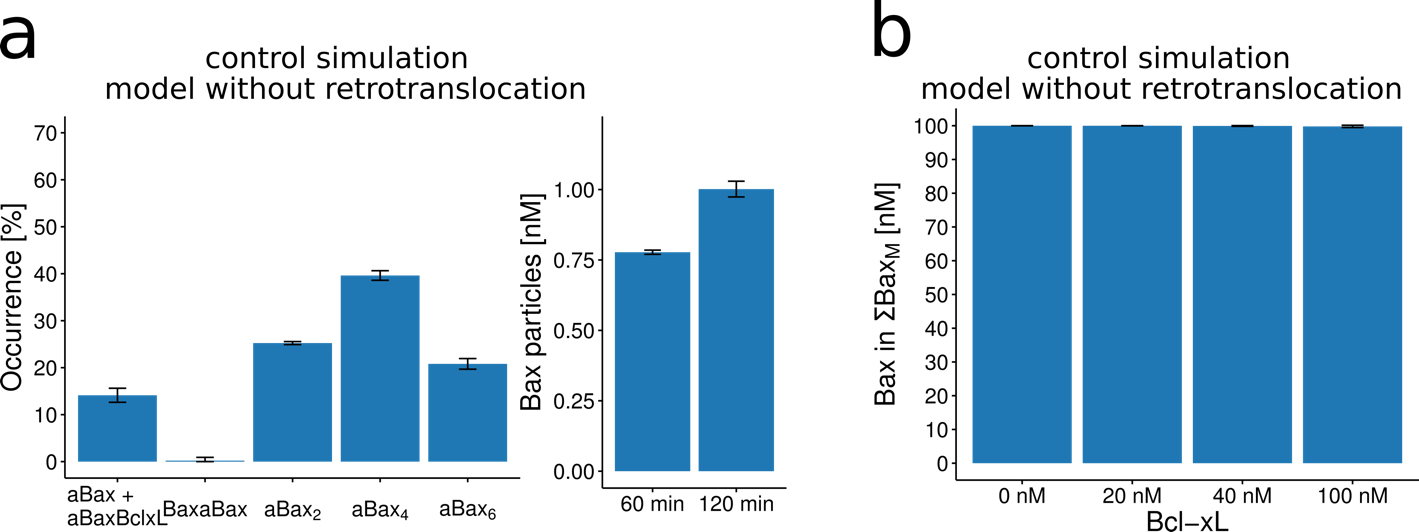
 **Supplementary Figure 1 a,** Control simulation of Bax oligomer distribution obtained from the model as parameterized in Figure 3e without accounting for Bcl-xL mediated retrotranslocation. **b**, Control simulation of tBid-induced ΣBax_M_ obtained from the model as parameterized in Figure 4c without accounting for Bcl-xL mediated retrotranslocation.


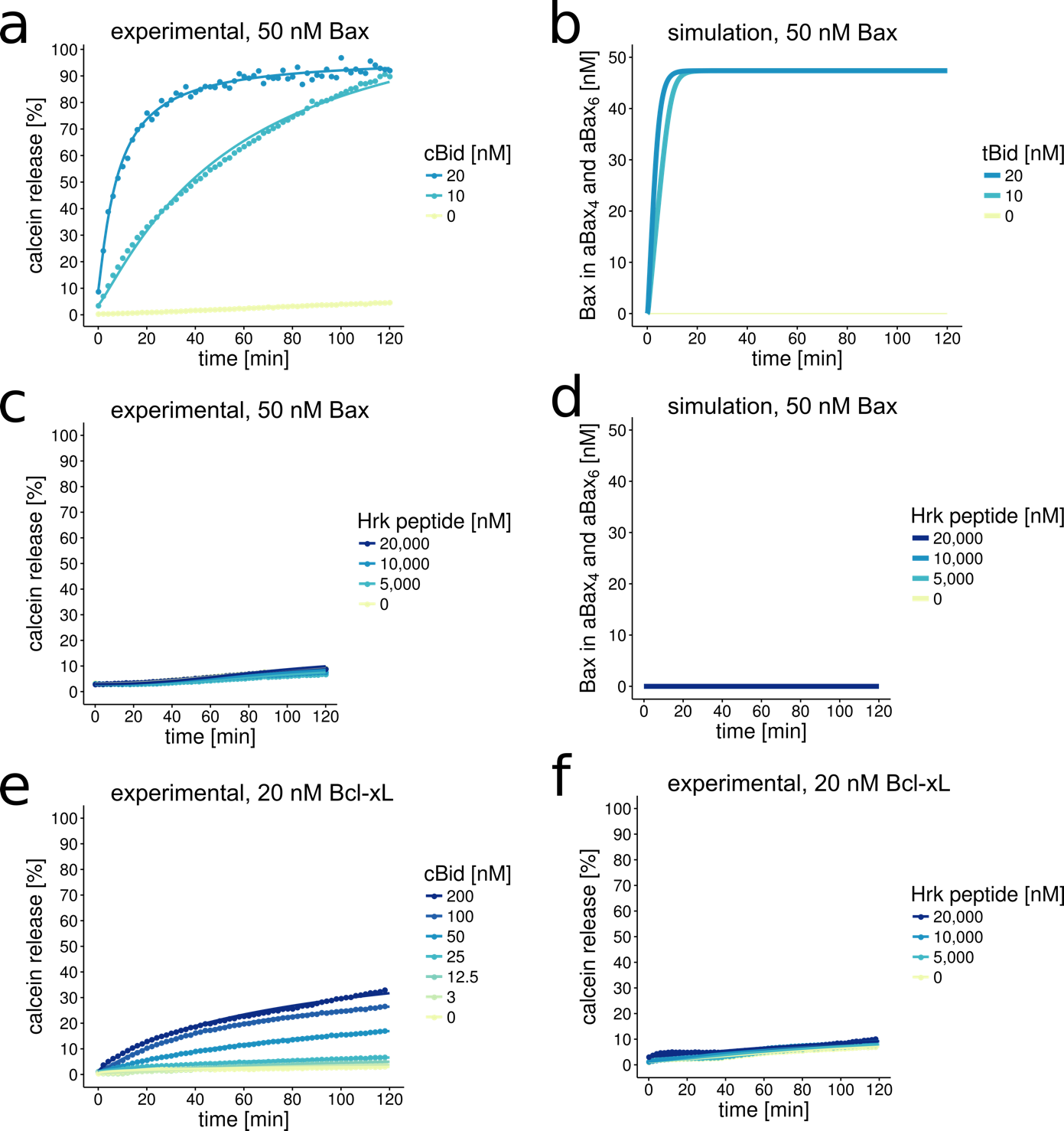
 **Supplementary Figure 2** Time courses of experimentally measured calcein release from large unilamellar vesicles (**a,c,e,f**) and modeled Bax recruitment into pores (**b,d**). **a,c** Calcein release as measured fro 50 nM Bax and varying amounts of cBid or Hrk peptide, respectively. **b,d** Simulation for 50 nM Bax and varying amounts of tBid or Hrk peptide, respectively. **e,f**, Calcein release as measured for 20 nM Bcl-xL and varying amounts of cBid or Hrk peptide, respectively.


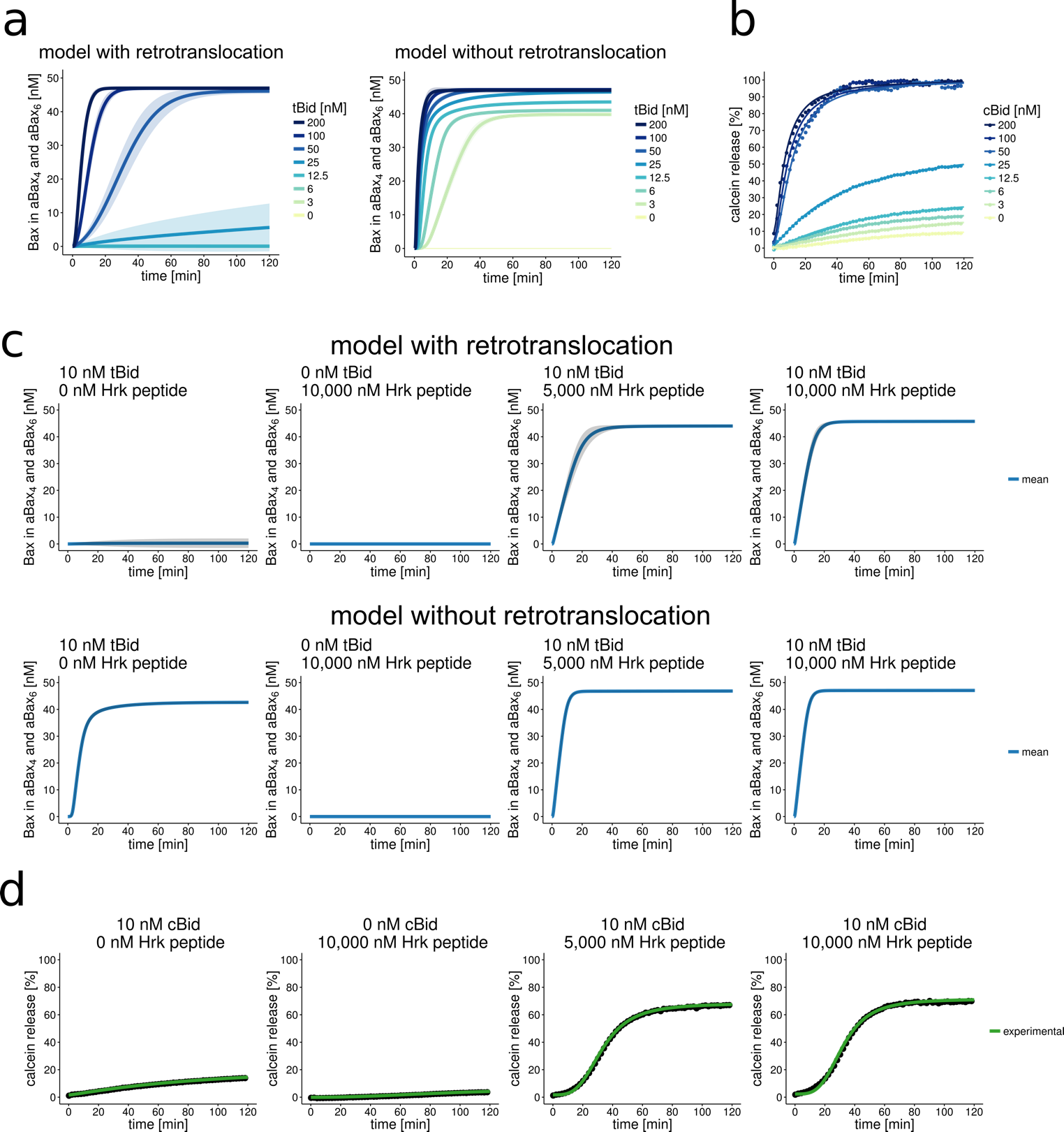


**Supplementary Figure 3** Time courses of modeled Bax recruitment into pores (**a,c**) and experimentally measured calcein release from large unilamellar vesicles (**b,d**). **a**, Simulations for 50 nM Bax with 20 nM Bcl-x_L_ and varying amounts of tBid, with and without accounting for retrotranslocation in the model. **b**, Calcein release as measured for 50 nM Bax, 20 nM Bcl-x_L_ and varying amounts of cBid. **c,d** Time courses of simulation and experimental measurements corresponding to simulations shown in Fig. 5e,f. Shaded areas in simulation plots correspond to SD of ensemble predictions, lines correspond to the means.


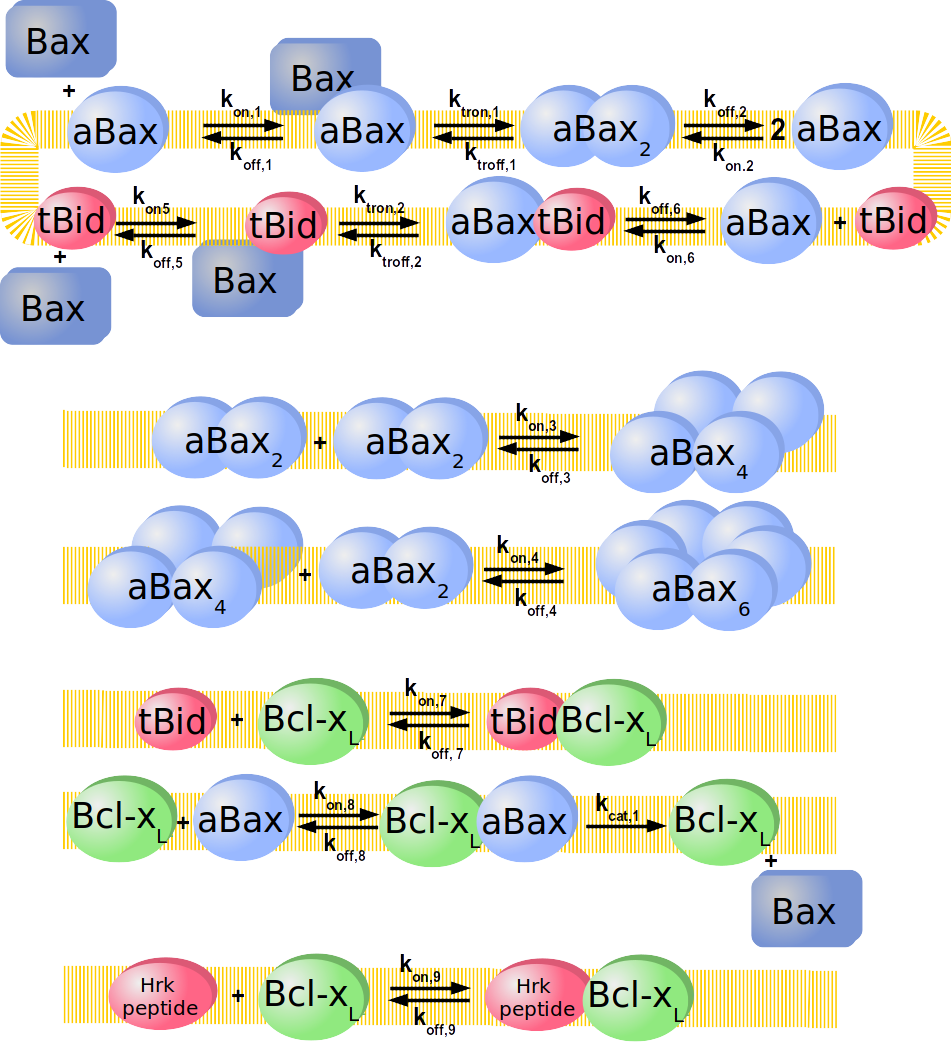


**Supplementary Figure 4** Overview of all interactions, translocations into and from the membrane to the aqueous phase, as well as retrotranslocation process that were integrated in the model. Numbers of rate constants serve as identifiers for the supplementary tables.

**Prior estimates of parameter values**

Biologically plausible parameter ranges were chosen within which subsequent model training was performed. K_D_ values were assumed to lie within 0.1 – 1000 nM, as affinity constants for interactions within the Bcl-2 family were reported in the low to high nM range (1). For k_on_ rate constants, values between 1e^-6^ – 1 nM^-1^s^-1^ were assumed to be reasonable ranges for protein-protein associations (2). k_off_ values were calculated from k_off_ = k_on_*K_D_.

Insertion of Bax into the membrane or release back into the cytosol was assumed with rates of 1e^-5^ - 1e^-1^ s^-1^. Retrotranslocation of Bax by Bcl-x_L_ was assumed to be within 1e^-5^ – 1e^-1^ s^-1^. For all of these processes, rate constants of the isolated reactions are unavailable and due to sparse information about the velocity of such processes, parameters spanning several orders of magnitude and approximately in the same range as k_off_ constants and the effective rate constant of Bax retrotranslocation in living cells (3) were assumed.

**Supplementary Table 1** Parameter ranges after model training used for simulations in the respective Figures. k_off_ values were calculated from K_D_ and k_on_. K_D_ values are given in nM, k_on_ in nM^-1^s^-1^


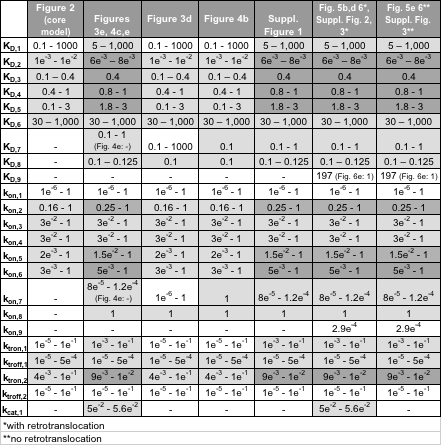

$$\text{with k}_{\text{off,x}}\text{ = }\text{K}_{\text{D,x}}\text{ ∙ }\text{k}_{\text{on,x}}$$

**Supplementary Table 2** Protein concentrations at t = 0 min (t_0_) used for simulations in the respective Figures. All (hetero- and homo-) dimers, tetramers, hexamers were considered to be absent at time t_0_ (0 nM).

|  | **Figure 2a,b** | **Figure 2c** | **Figure 3,**  **Suppl. Fig. 1a** | **Figure 4,**  **Suppl. Fig. 1b** | **Figure 5b,d,e**  **Suppl. Fig. 3** | **Fig. 6** | **Suppl. Fig. 2** |
| --- | --- | --- | --- | --- | --- | --- | --- |
| **Bax [nM]** | 2.5 | 2.5 - aBax | 2.5 | 100 | 50 | 50 | 50 |
| **aBax [nM]** | - | varying | - | - | - | - | - |
| **tBid [nM]** | 5 | - | 5 | 20 | varying | 10 | varying |
| **Bcl-xL [nM]** | - | - | 2.5 (at t_60_) | 0/20/40/100 | 20 | varying | - |
| **Hrk peptide [nM]** | - | - | - | - | varying | 0/5,000/50 | varying |
| **simulation [min]** | 5/10/60 | 60 | 120 | 120 | 120 | 120 | 120 |

**Supplementary Table 3** Definition of outputs of simulations presented in the respective Figures.

| **Fig.** | **Output** | **Unit** | **Formula** | **If applicable: as % of** |
| --- | --- | --- | --- | --- |
| 2 | aBax | % | aBax + aBaxtBid | aBax + aBaxtBid + 2 BaxaBax + 2 aBax_2_ + 4 aBax4 + 6 aBax_6_ |
| 2 | BaxaBax | % | 2 BaxaBax | aBax + aBaxtBid + 2 BaxaBax + 2 aBax_2_ + 4 aBax4 + 6 aBax_6_ |
| 2 | aBax_2_ | % | 2 aBax_2_ | aBax + aBaxtBid + 2 BaxaBax + 2 aBax_2_ + 4 aBax4 + 6 aBax_6_ |
| 2 | aBax_4_ | % | 4 aBax_4_ | aBax + aBaxtBid + 2 BaxaBax + 2 aBax_2_ + 4 aBax4 + 6 aBax_6_ |
| 2 | aBax_6_ | % | 6 aBax_6_ | aBax + aBaxtBid + 2 BaxaBax + 2 aBax_2_ + 4 aBax4 + 6 aBax_6_ |
| 3, S1a | aBax + aBaxBclxL | % | aBax + aBaxtBid + aBaxBclxL | aBax + aBaxtBid + 2 BaxaBax + 2 aBax_2_ + 4 aBax4 + 6 aBax_6_ + aBaxBclxL |
| 3, S1a | BaxaBax | % | 2 BaxaBax | aBax + aBaxtBid + 2 BaxaBax + 2 aBax_2_ + 4 aBax4 + 6 aBax_6_ + aBaxBclxL |
| 3, S1a | aBax_2_ | % | 2 aBax_2_ | aBax + aBaxtBid + 2 BaxaBax + 2 aBax_2_ + 4 aBax4 + 6 aBax_6_ + aBaxBclxL |
| 3, S1a | aBax_4_ | % | 4 aBax_4_ | aBax + aBaxtBid + 2 BaxaBax + 2 aBax_2_ + 4 aBax4 + 6 aBax_6_ + aBaxBclxL |
| 3, S1a | aBax_6_ | % | 6 aBax_6_ | aBax + aBaxtBid + 2 BaxaBax + 2 aBax_2_ + 4 aBax4 + 6 aBax_6_ + aBaxBclxL |
| 3, S1a | Bax particles | nM | aBax + BaxaBax + aBax_2_ + aBax_4_ + aBax_6_ + tBidaBax + BclxLaBax | - |
| 4, S1b | ΣBax_M_ | nM | aBax + aBaxtBid + 2 BaxaBax + 2 aBax_2_ + 4 aBax4 + 6 aBax_6_ + aBaxBclxL + BaxtBid | - |
| 5,6, S2, S3 | Bax in aBax_4_ and aBax_6_ | nM | 4 aBax4 + 6 aBax_6_ | - |

**Analysis of parameter ranges of trained core model and model with Bcl-x_L_ retrotranslocation activity**

Shown are simulation results for all parameters whose values were restricted in the model training procedure. Each dot in the following dot plots corresponds to one model simulation. Black vertical lines correspond to boundaries of the parameter range after model training. Shown are the simulation results of outputs for which experimental data were available (such as the occurrence aBax_2_ in %) and which implicated a restriction of the respective parameter to train the model.

Please note that for Supplementary Figure 5-16, we omitted very early time points from training data sets taken from (4) to avoid overfitting of the ODE-model to conditions where reaction processes might still be influenced substantially by stochasticity. Indeed, inclusion of such data would result in excessively fast and unjustifiable rates for tBid-induced Bax membrane integration in the order of 0.3-1 s^-1^ (not shown) that would contradict experimental observations that indicate Bax membrane insertion to be the rate-limiting step of tBid-mediated activation (5).


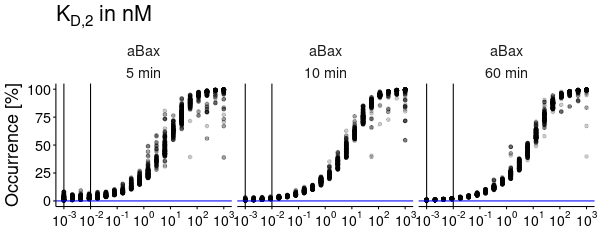


**Supplementary Figure 5** Simulation results using the starting conditions as in Figure 2a,b and readout at the indicated time points. Shown are sampled parameterizations across the original assumed plausible range of parameter K_D,2_ of the core model (before model training). Blue horizontal lines indicate experimentally valid range for the respective model outputs as estimated from original publication (4).


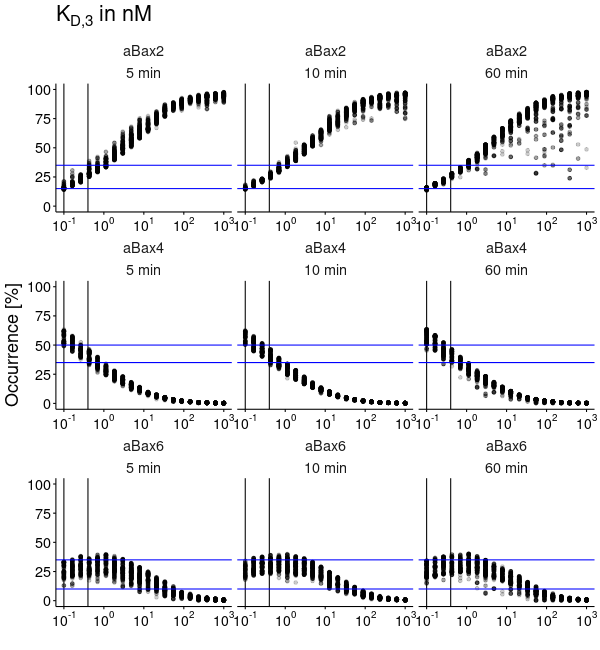


**Supplementary Figure 6** Simulation results using the starting conditions as in Figure 2a,b and readout at the indicated time points. Shown are sampled parameterizations across the original assumed plausible range of parameter K_D,3_ of the core model (before model training). Blue horizontal lines indicate experimentally valid range for the respective model outputs as estimated from original publication (4).


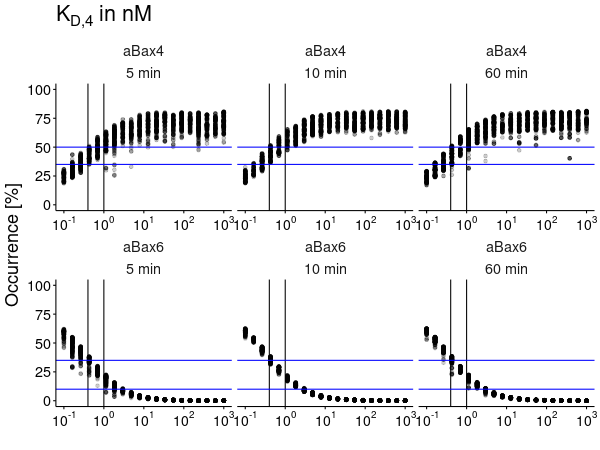


**Supplementary Figure 7** Simulation results using the starting conditions as in Figure 2a,b and readout at the indicated time points. Shown are sampled parameterizations across the original assumed plausible range of parameter K_D,4_ of the core model (before model training). Blue horizontal lines indicate experimentally valid range for the respective model outputs as estimated from original publication (4).


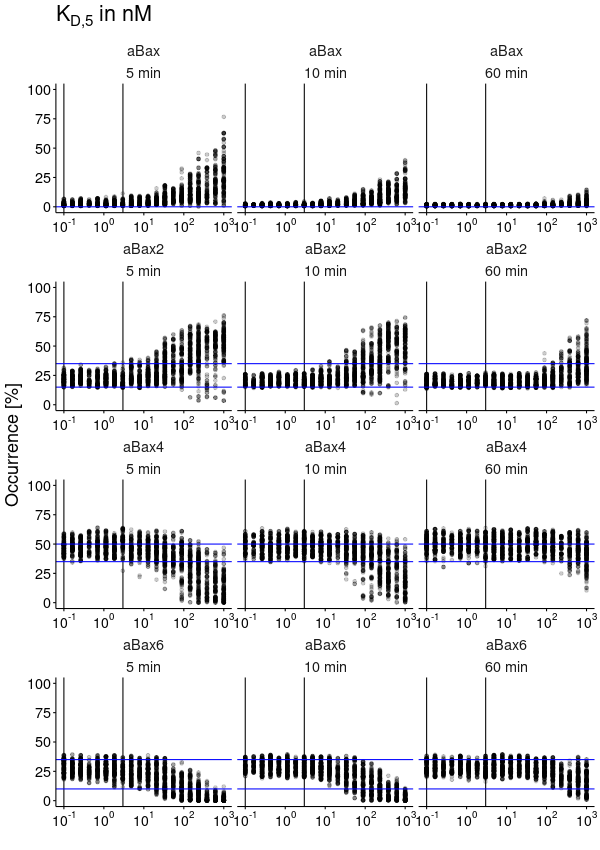


**Supplementary Figure 8** Simulation results using the starting conditions as in Figure 2a,b and readout at the indicated time points. Shown are sampled parameterizations across the original assumed plausible range of parameter K_D,5_ of the core model (before model training). Blue horizontal lines indicate experimentally valid range for the respective model outputs as estimated from original publication (4).


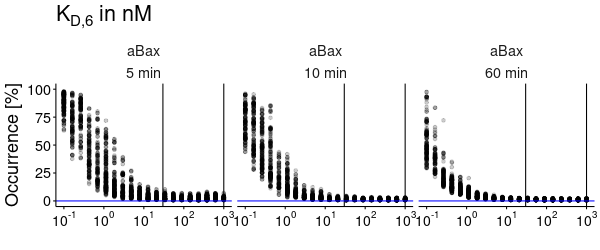


**Supplementary Figure 9** Simulation results using the starting conditions as in Figure 2a,b and readout at the indicated time points. Shown are sampled parameterizations across the original assumed plausible range of parameter K_D,6_ of the core model (before model training). Blue horizontal lines indicate experimentally valid range for the respective model outputs as estimated from original publication (4).


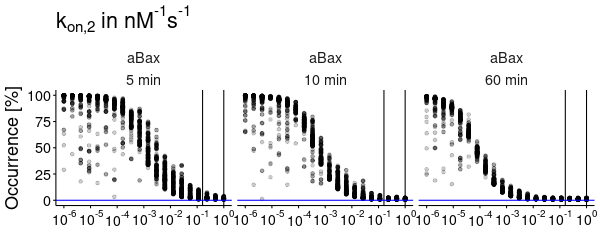


**Supplementary Figure 10** Simulation results using the starting conditions as in Figure 2a,b and readout at the indicated time points. Shown are sampled parameterizations across the original assumed plausible range of parameter k_on,2_ of the core model (before model training). Blue horizontal lines indicate experimentally valid range for the respective model outputs as estimated from original publication (4).


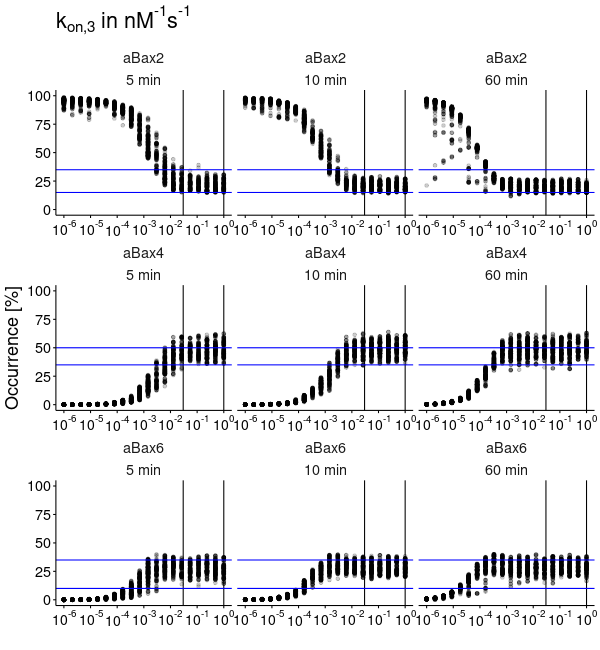


**Supplementary Figure 11** Simulation results using the starting conditions as in Figure 2a,b and readout at the indicated time points. Shown are sampled parameterizations across the original assumed plausible range of parameter k_on,3_ of the core model (before model training). Blue horizontal lines indicate experimentally valid range for the respective model outputs as estimated from original publication (4).


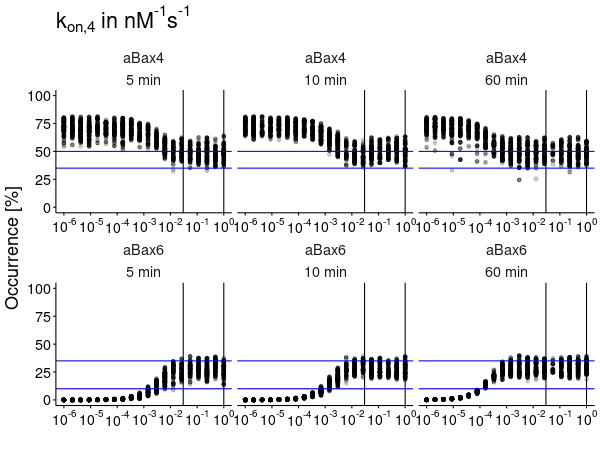


**Supplementary Figure 12** Simulation results using the starting conditions as in Figure 2a,b and readout at the indicated time points. Shown are sampled parameterizations across the original assumed plausible range of parameter k_on,4_ of the core model (before model training). Blue horizontal lines indicate experimentally valid range for the respective model outputs as estimated from original publication (4).


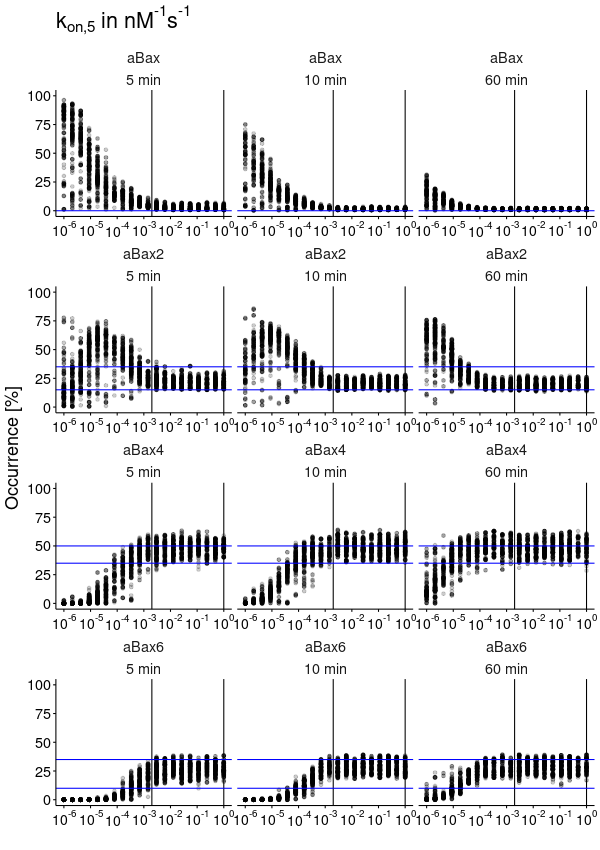


**Supplementary Figure 13** Simulation results using the starting conditions as in Figure 2a,b and readout at the indicated time points. Shown are sampled parameterizations across the original assumed plausible range of parameter k_on,5_ of the core model (before model training). Blue horizontal lines indicate experimentally valid range for the respective model outputs as estimated from original publication (4).


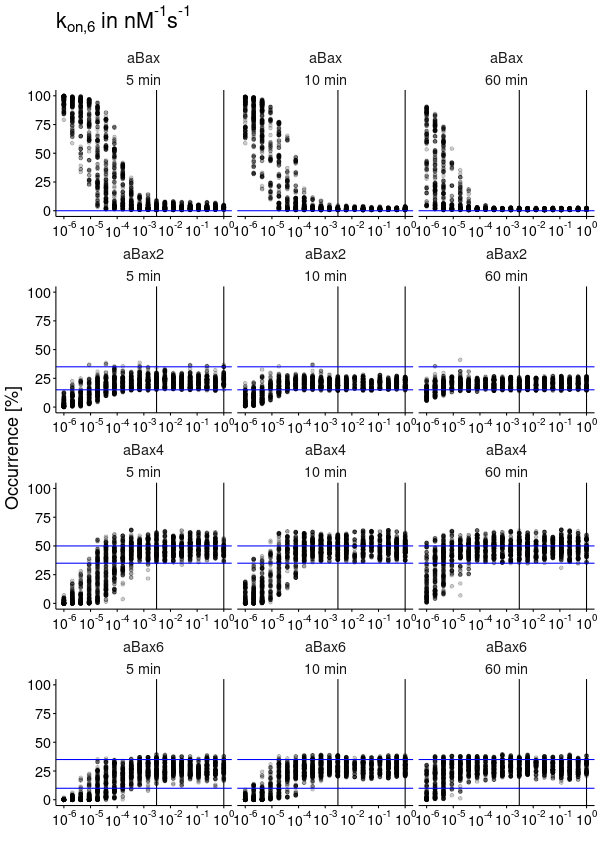


**Supplementary Figure 14** Simulation results using the starting conditions as in Figure 2a,b and readout at the indicated time points. Shown are sampled parameterizations across the original assumed plausible range of parameter k_on,6_ of the core model (before model training). Blue horizontal lines indicate experimentally valid range for the respective model outputs as estimated from original publication (4).


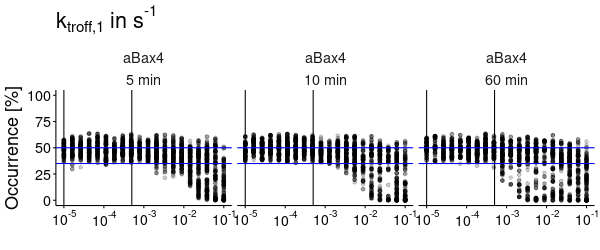


**Supplementary Figure 15** Simulation results using the starting conditions as in Figure 2a,b and readout at the indicated time points. Shown are sampled parameterizations across the original assumed plausible range of parameter k_troff,1_ of the core model (before model training). Blue horizontal lines indicate experimentally valid range for the respective model outputs as estimated from original publication (4).


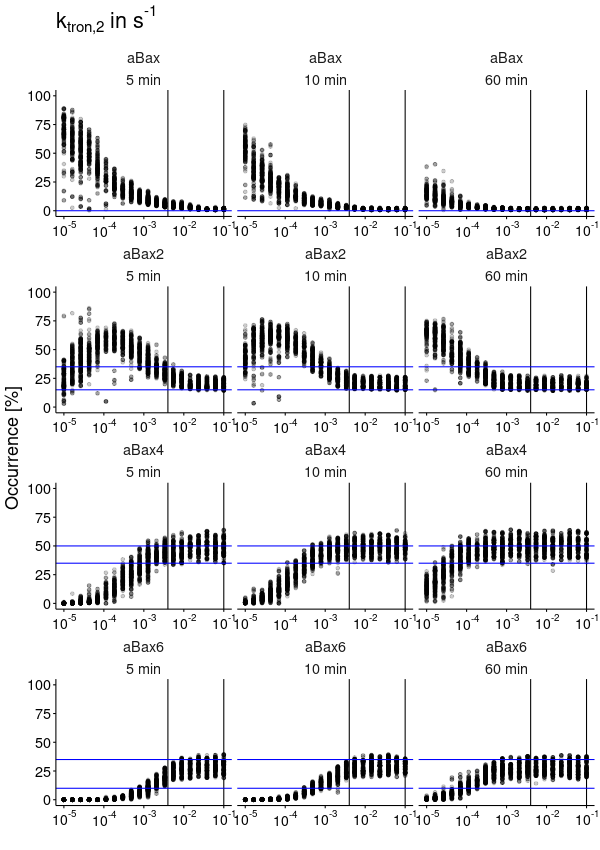


**Supplementary Figure 16** Simulation results using the starting conditions as in Figure 2a,b and readout at the indicated time points. Shown are sampled parameterizations across the original assumed plausible range of parameter k_tron,2_ of the core model (before model training). Blue horizontal lines indicate experimentally valid range for the respective model outputs as estimated from original publication (4).


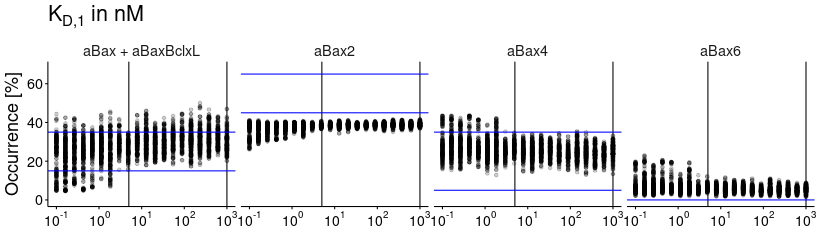


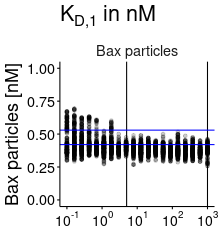


**Supplementary Figure 17** Simulation results using the starting conditions as in Figure 3e. Shown are sampled parameterizations across the parameter range of the core model of parameter K_D,1_. Blue horizontal lines indicate experimentally valid range for the respective model outputs as estimated from original publication (4).


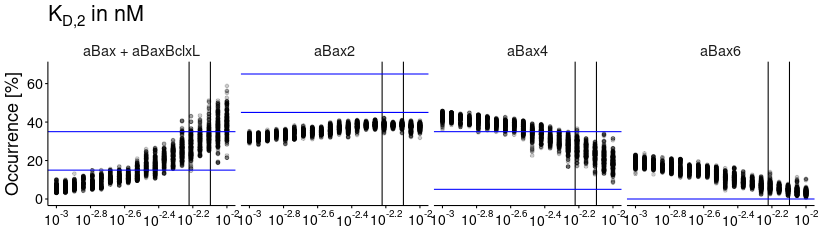


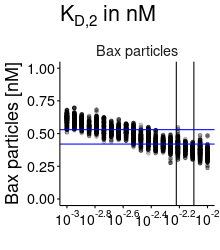


**Supplementary Figure 18** Simulation results using the starting conditions as in Figure 3e. Shown are sampled parameterizations across the parameter range of the core model of parameter K_D,2_. Blue horizontal lines indicate experimentally valid range for the respective model outputs as estimated from original publication (4).

_
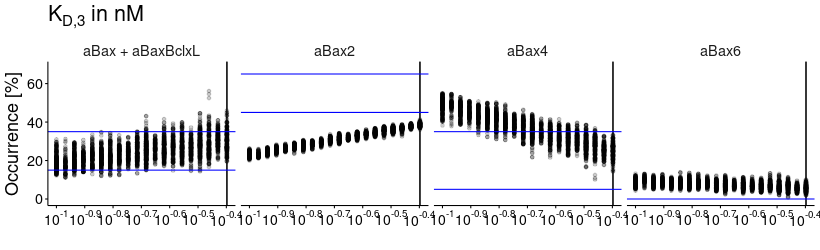
_

**
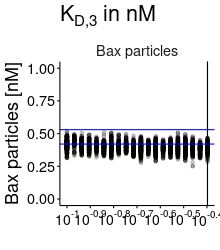
**

**Supplementary Figure 19** Simulation results using the starting conditions as in Figure 3e. Shown are sampled parameterizations across the parameter range of the core model of parameter K_D,3_. Blue horizontal lines indicate experimentally valid range for the respective model outputs as estimated from original publication (4).

_
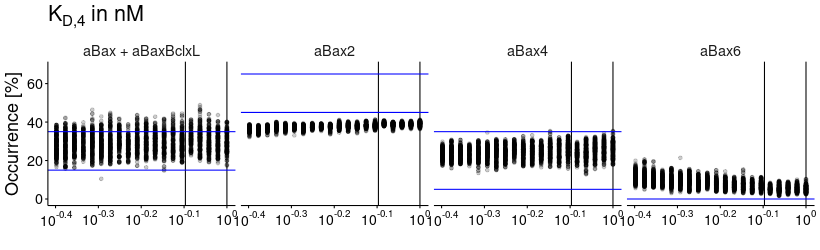
_

**
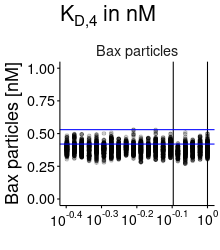
**

**Supplementary Figure 20** Simulation results using the starting conditions as in Figure 3e. Shown are sampled parameterizations across the parameter range of the core model of parameter K_D,4_. Blue horizontal lines indicate experimentally valid range for the respective model outputs as estimated from original publication (4).

_
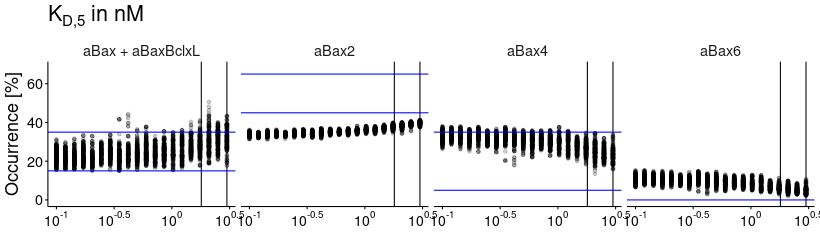
_

**
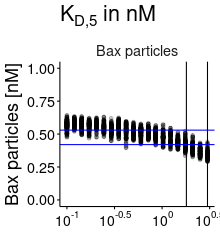
**

**Supplementary Figure 21** Simulation results using the starting conditions as in Figure 3e. Shown are sampled parameterizations across the parameter range of the core model of parameter K_D,5_. Blue horizontal lines indicate experimentally valid range for the respective model outputs as estimated from original publication (4).


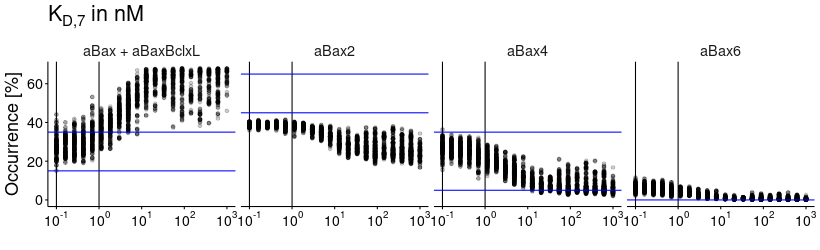


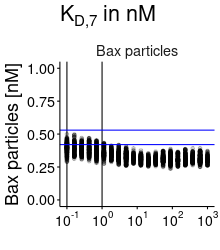


**Supplementary Figure 22** Simulation results using the starting conditions as in Figure 3e. Shown are sampled parameterizations across the parameter range of the core model of parameter K_D,7_. Blue horizontal lines indicate experimentally valid range for the respective model outputs as estimated from original publication (4).


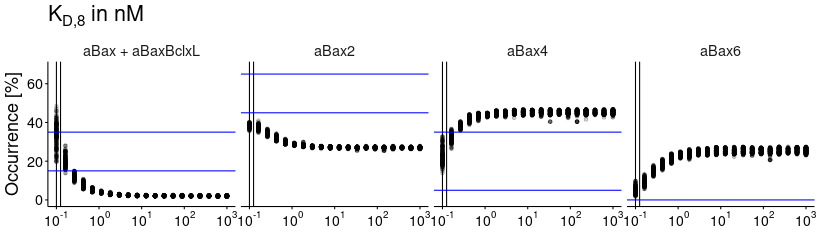


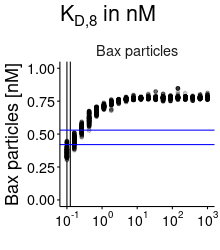


**Supplementary Figure 23** Simulation results using the starting conditions as in Figure 3e. Shown are sampled parameterizations across the parameter range of the core model of parameter K_D,8_. Blue horizontal lines indicate experimentally valid range for the respective model outputs as estimated from original publication (4).


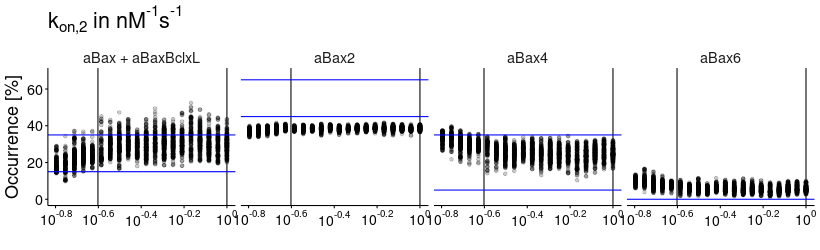


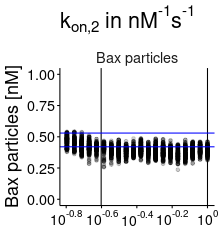


**Supplementary Figure 24** Simulation results using the starting conditions as in Figure 3e. Shown are sampled parameterizations across the parameter range of the core model of parameter k_on,2_. Blue horizontal lines indicate experimentally valid range for the respective model outputs as estimated from original publication (4).


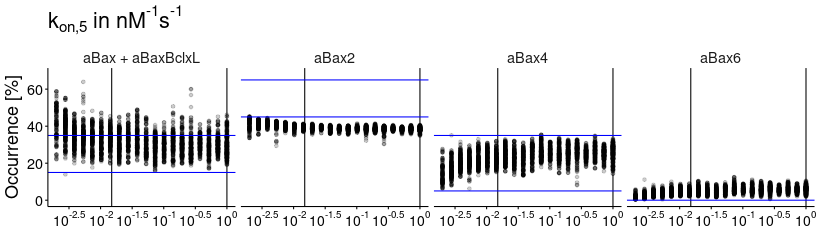


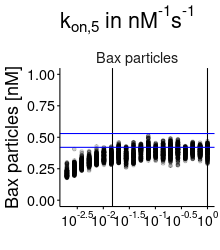


**Supplementary Figure 25** Simulation results using the starting conditions as in Figure 3e. Shown are sampled parameterizations across the parameter range of the core model of parameter k_on,5_. Blue horizontal lines indicate experimentally valid range for the respective model outputs as estimated from original publication (4).


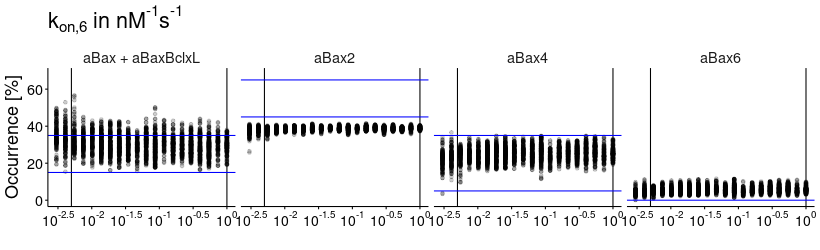


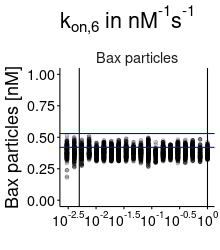


**Supplementary Figure 26** Simulation results using the starting conditions as in Figure 3e. Shown are sampled parameterizations across the parameter range of the core model of parameter k_on,6_. Blue horizontal lines indicate experimentally valid range for the respective model outputs as estimated from original publication (4).


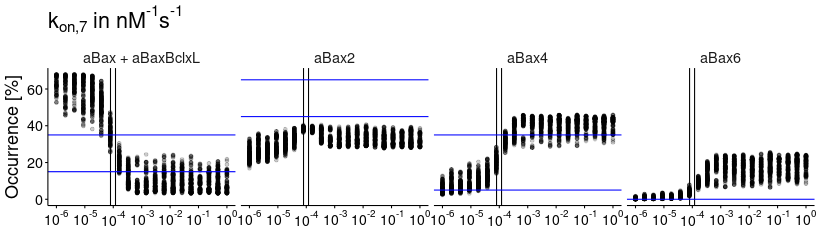


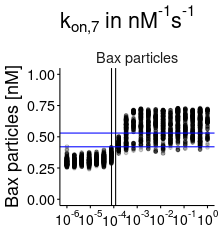


**Supplementary Figure 27** Simulation results using the starting conditions as in Figure 3e. Shown are sampled parameterizations across the parameter range of the core model of parameter k_on,7_. Blue horizontal lines indicate experimentally valid range for the respective model outputs as estimated from original publication (4).


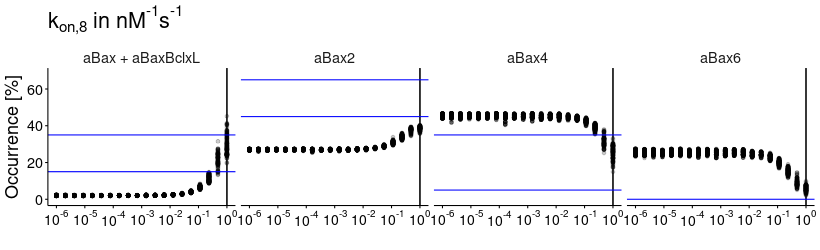


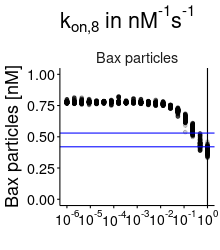


**Supplementary Figure 28** Simulation results using the starting conditions as in Figure 3e. Shown are sampled parameterizations across the parameter range of the core model of parameter k_on,8_. Blue horizontal lines indicate experimentally valid range for the respective model outputs as estimated from original publication (4).


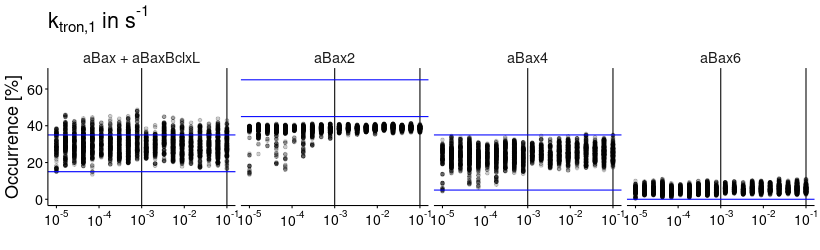


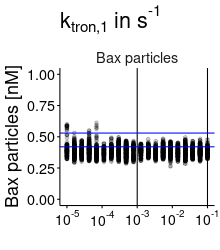


**Supplementary Figure 29** Simulation results using the starting conditions as in Figure 3e. Shown are sampled parameterizations across the parameter range of the core model of parameter k_tron,1_. Blue horizontal lines indicate experimentally valid range for the respective model outputs as estimated from original publication (4).

_
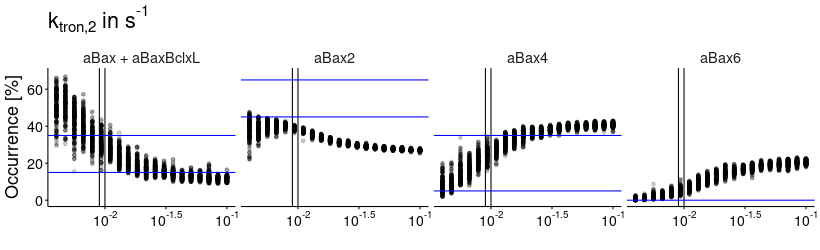
_

**
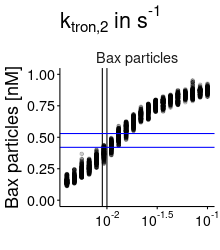
**

**Supplementary Figure 30** Simulation results using the starting conditions as in Figure 3e. Shown are sampled parameterizations across the parameter range of the core model of parameter k_tron,2_. Blue horizontal lines indicate experimentally valid range for the respective model outputs as estimated from original publication (4).


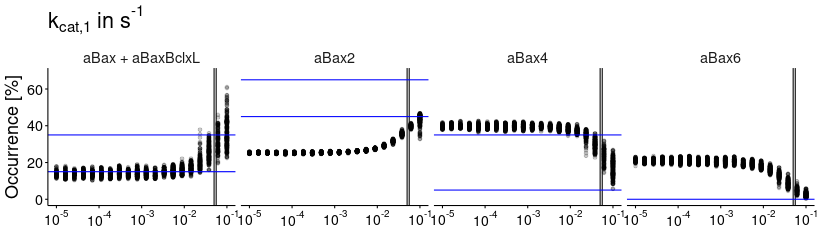


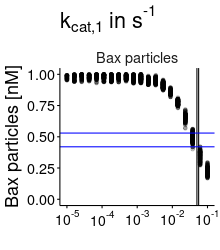


**Supplementary Figure 31** Simulation results using the starting conditions as in Figure 3e. Shown are sampled parameterizations across the parameter range of the core model of parameter k_cat,1_. Blue horizontal lines indicate experimentally valid range for the respective model outputs as estimated from original publication (4).

**Bibliography**

1. Hantusch A, Brunner T, Frickey T, Rehm M. Bcl-2-Ome – a database and interactive web service for dissecting the Bcl-2 interactome. Cell Death Differ. 2017 Jan;24(1):192–192.

2. Schreiber G, Haran G, Zhou H-X. Fundamental Aspects of Protein−Protein Association Kinetics. Chem Rev. 2009 Mar 11;109(3):839–60.

3. Edlich F, Banerjee S, Suzuki M, Cleland MM, Arnoult D, Wang C, et al. Bcl-xL Retrotranslocates Bax from the Mitochondria into the Cytosol. Cell. 2011 Apr;145(1):104–16.

4. Subburaj Y, Cosentino K, Axmann M, Pedrueza-Villalmanzo E, Hermann E, Bleicken S, et al. Bax monomers form dimer units in the membrane that further self-assemble into multiple oligomeric species. Nat Commun. 2015 Aug 14;6:8042.

5. Lovell JF, Billen LP, Bindner S, Shamas-Din A, Fradin C, Leber B, et al. Membrane binding by tBid initiates an ordered series of events culminating in membrane permeabilization by Bax. Cell. 2008 Dec 12;135(6):1074–84.
